# Socialization of Children with Special Educational Needs (SEN)

**DOI:** 10.1192/j.eurpsy.2026.11835

**Published:** 2026-07-31

**Authors:** A. Akhmetzyanova, S. Akhmetzianov

**Affiliations:** 1 Department of Psychology and Pedagogy of Special Education, Kazan Federal University, Kazan, Russian Federation; 2 English Language Department, Pennsylvania American School System, Ho Chi Minh, Viet Nam

## Abstract

**Introduction:**

The key task of education is the development of personal qualities that contribute to the socialization of the child, allowing him to independently determine behavior strategies, compare his own behavior with social norms, and the expectations of partners in the interaction situation.

**Objectives:**

identifying patterns that make it possible to develop skills that promote the socialization of children with **SEN** without resorting to a comprehensive transformation of the educational process in an inclusive educational institution.

**Methods:**

The study involved 100 preschool children **with special educational needs**. The corrective and developmental work was carried out using the “Prognostic Stories” technique developed.

**Results:**

The following patterns of socialization of children with special educational needs were identified:

The space of socialization as a system of significant relationships with others is determined by the partner in the relationship with whom the preschooler’s interaction is realized.

The effectiveness of socialization for children in an inclusive educational environment is determined by the development of the child’s forecasting skills.

Promoting socialization will be effective when implementing a gradual transition from social interaction situations that are familiar to the child to situations in which the child has limited experience of interaction.

Indicators of the effectiveness of socialization are the child’s forecasting skills, the assessment of which can be carried out according to the following criteria: identification of a social norm; optimistic attitude; construction of an active position; variability of forecasts; forecasting long-term results; realistic images of the future situation; ability to use speech.

The results of the study demonstrated significant qualitative changes, which manifested themselves in the activation of interest in interaction, children began to rely on the help of an adult, and began to control their behavior and emotions somewhat more.

**Conclusions:**

Correctional and developmental work using the “Predictive Stories” technology helps to include SEN children in the environment of their neurotypical peers. The “Prognostic Stories” technique creates conditions for developing the social interaction skills of children with special educational needs with participants in the inclusive educational environment, thereby increasing the effectiveness of socialization.

**Disclosure of Interest:**

None Declared

